# Global impacts of heat and water stress on food production and severe food insecurity

**DOI:** 10.1038/s41598-024-65274-z

**Published:** 2024-06-22

**Authors:** Tom Kompas, Tuong Nhu Che, R. Quentin Grafton

**Affiliations:** 1 Centre of Excellence for Biosecurity Risk Analysis and the Centre for Environmental and Economic Research, School of Agriculture, Food and Ecosystem Sciences, Faculty of Science, University of Melbourne, Melbourne, Australia; 2 Global Environmental and Economic Modelling, Canberra, Australia; 3 Crawford School of Public Policy, Australian National University, Canberra, Australia

**Keywords:** Water stress, Climate change, Agricultural productivity, Food security, Irrigation, Computable General Equilibrium (CGE) models, Environmental economics, Climate-change impacts, Projection and prediction

## Abstract

In contrast to most integrated assessment models, with limited transparency on damage functions and recursive temporal dynamics, we use a unique large-dimensional computational global climate and trade model, GTAP-DynW, to directly project the possible intertemporal impacts of water and heat stress on global food supply and food security to 2050. The GTAP-DynW model uses GTAP production and trade data for 141 countries and regions, with varying water and heat stress baselines, and results are aggregated into 30 countries/regions and 30 commodity sectors. Blue water stress projections are drawn from WRI source material and a GTAP-Water database to incorporate dynamic changes in water resources and their availability in agricultural production and international trade, thus providing a more general measure for severe food insecurity from water and heat stress damages with global warming. Findings are presented for three representative concentration pathways: RCP4.5-SSP2, RCP8.5-SPP2, and RCP8.5-SSP3 (population growth only for SSPs) and project: (a) substantial declines, as measured by GCal, in global food production of some 6%, 10%, and 14% to 2050 and (b) the number of additional people with severe food insecurity by 2050, correspondingly, increases by 556 million, 935 million, and 1.36 billion compared to the 2020 model baseline.

Climate change is a serious threat to food production systems that are highly dependent on water resources and ecosystems, at multiple scales^1^. Various regions already suffer from water cycle disruptions due to climate change which include intensification of extreme weather events (e.g., droughts, floods) and groundwater depletion^2–4^. Critical future risks include heat stress and water stress on global food production and, thus, food security^5^. Climate change risks are magnified by increasing water withdrawals for household and industry to 2050^6^, especially for irrigated agriculture that accounts for about 70% of total water withdrawals, and supplies up to 40% of the global human-consumed calories^7^.

How these risks are realized, when and where, is determined by domestic and international input–output interactions across commodity sectors and regions, and endowments such as capital, labor, land, natural resources, and water, and the interlinkages of international trade. To quantitatively assess these risks for global food production and food security requires a Computable General Equilibrium (CGE) model, connected to a climate change model, to capture price, trade, and income effects in relation to both food supply and demand.

Previous integrated assessment model (IAM) studies simulating the Shared Socioeconomic Pathways (SSPs) find that changes in population growth and dietary makeup, as well as agricultural efficiencies, lead to increased food insecurity under SSP3 and SSP4^8^. Further, mitigation efforts aimed at lowering land-based emissions have the effect of increasing the cost of production and consequently food prices, particularly within low-income regions^9,10^. Consequently, SSP top-down results need additional variants to account for inequity in food availability and accessibility^11^.

In contrast to the vast majority of extant IAM studies that employ coupled-model frameworks with recursive temporal dynamics and with limited transparency about damage functions, our analysis circumvents the need for model coupling through the incorporation of climate change damages and agricultural dynamics, such as changes in irrigation, directly into the core model. In conjunction with intertemporal dynamics, our approach allows for the simulation of feedbacks directly into the model solution with optimization taking place across all time steps simultaneously.^12^ Further, the current study implements a wider range of climate change damages on factors of production, such as the impact of heat stress on labor productivity, which is frequently missing within IAM.

We used a unique intertemporal CGE model, GTAP-DynW, that extends the GTAP-AEZ, Version 10^13^ platform, and includes the GTAP-Water dataset^14^ to project global food production and food security to 2050. GTAP-DynW incorporates dynamic changes in water resources and their availability in agricultural production and international trade and also includes a food security component with climate change damages^15–17^. Model results are aggregated from 141 countries in GTAP-DynW to 30 countries and/or regions and for 30 commodities to provide global projections.

The scenario framework adopted here is based on SSP quantitative demographic data^18^ from the SSP2 and SSP3 scenarios as well as temperature pathways associated with the Representative Concentration Pathway (RCP) RCP4.5 and RCP8.5. Note that the current model does not directly encompass emissions and, therefore, combinations deemed infeasible within a standard SSP-RCP framework (e.g., SSP2-RCP8.5) are a potential future outcome due to uncertainties regarding feedbacks, and climate sensitivity^19,20^. For each of the three reported projections, we used the same population projection for each SSP (2,3) and ensured the temperature pathways in our model were consistent with the given RCP (4.5, 8.5). We assumed that the RCP8.5 scenario to 2100 remains useful in terms of model runs which use a mid-century time horizon^21^.

The impacts of water and heat stress from climate change on agricultural production and food security to 2050 were quantified using GTAP-DynW for three SSP population (only)/temperature combinations, SSP2-RCP4.5, SSP2-RCP8.5, and SSP3-RCP8.5 for decadal projections to 2050. These combinations are consistent with the WRI and GTAP databases. Impacts on individual agricultural commodities (e.g., paddy rice, wheat, etc.) from both irrigated and non-irrigated agriculture were summarized as GCal measures of global food production by country/region^22^ relative to 2020. Estimates of the additional people by 2050, relative to 2020, who could be classified with severe food insecurity, as per the Food Insecurity Experience Scale^23^, were then calculated by dividing the projected global food supply in GCal by the average per person dietary requirements per year.

Detailed information on the estimates of the heat and water stress damage functions and all relevant source material are given in the Supplementary Information. The initial projections for water stress are at the basin level and estimated as the ratio of water withdrawals to available blue water. Technical detail on model construction of GTAP-DynW is in the "Methods" section below and an illustrative figure of model structure for GTAP-DynW is contained in the Supplementary Information. Note that our projections of water and heat stress apply only to irrigated agriculture. For non-irrigated agriculture we make projections based on heat stress alone.

## Results

Model results for SSP2-RCP4.5, SPP2-RCP8.5, and SSP3-RCP8.5, respectively, project: (a) substantial declines, as measured by GCal, in global food production of some 6%, 10%, and 14% to 2050 and (b) the number of additional people with severe food insecurity by 2050, correspondingly, increases by 556 million, 935 million, and 1.36 billion compared to the 2020 model baseline.

### Food production

Food production is aggregated as total nutrition by thousand Giga-calories (thous. GCal) by region and million Gcal for the world using nutritional conversion factors for the total production of food across all food commodity sectors. A decrease in agricultural output causes a reduction in global food production (measured in total energy for nutrition) that, in turn, increases the number of people (millions) with severe food insecurity. GTAP-DynW provides these measures as model output for all climate change scenarios.

**Figure 1:**
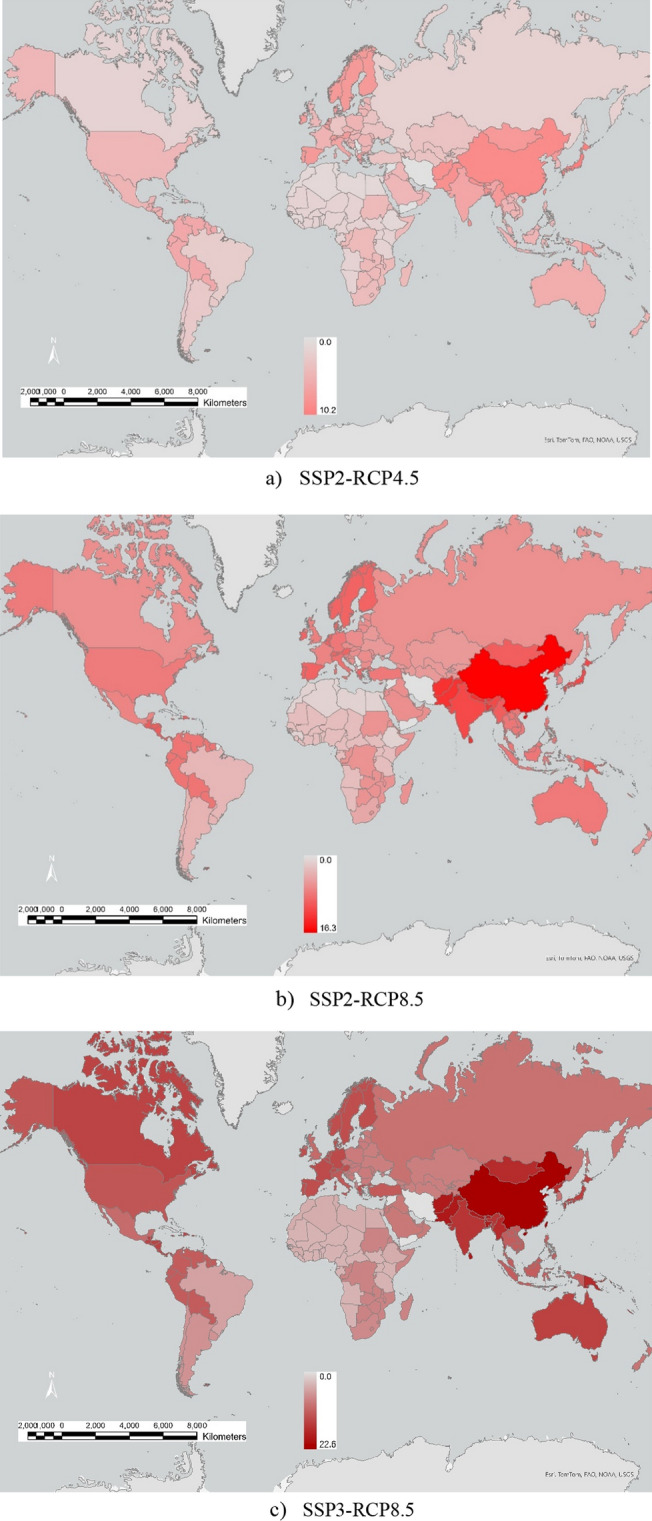
| Regional food production reduction from irrigated agriculture due to heat stress and water stress in 2050 relative 2020 *(% ranges).* Model output; maps generated using ArcGIS Pro 3.3 (https://www.esri.com).

Figure 1a–c shows a decreasing trend of food production as a % reduction in 2050 from 2020 for each climate change scenario. Global food production falls by 5.8%, 9.7%, and 14.2%, on average, for the scenarios SSP2-RCP4.5, SSP2-RCP8.5, and SSP3-RCP8.5, respectively. Globally, for scenarios SSP2-RCP4.5, SSP2-RCP8.5, and SSP3-RCP8.5, food production decreases from 9.75 million to 9.2, 8.8, and 8.4 million GCal, respectively by 2050.

For SSP2-RCP4.5 the food production from both water and heat stress is projected to fall by 5.1–6.6% in Africa, 5.8% in Australia, and 6.4% for some parts of South America. In 2050, food production is projected to fall by 4.8% for the USA, 9.0% for China, and 6.5% for India. For SSP3-RCP8.5, the worst-case scenario, food production is projected to decline by 8.2–11.8% in Africa, 14.7% for Australia, and 19.4% for some parts of Central America. For SSP3-RCP8.5, in 2050, global food production is projected to fall by 12.6% for the USA, 22.4% for China, and 16.1% for India.

### Food security

The number of persons (millions) with severe food insecurity caused by a decrease in global food production (or aggregated nutritional supply) is calculated as the reduction in food production relative to base nutritional supply. All outputs are trade-adjusted, noting that for net food exporting countries/regions a fall in their domestically produced food production does not necessarily increase their domestic food insecurity (e.g., Australia, France, Russia and the USA).

**Figure 2:**
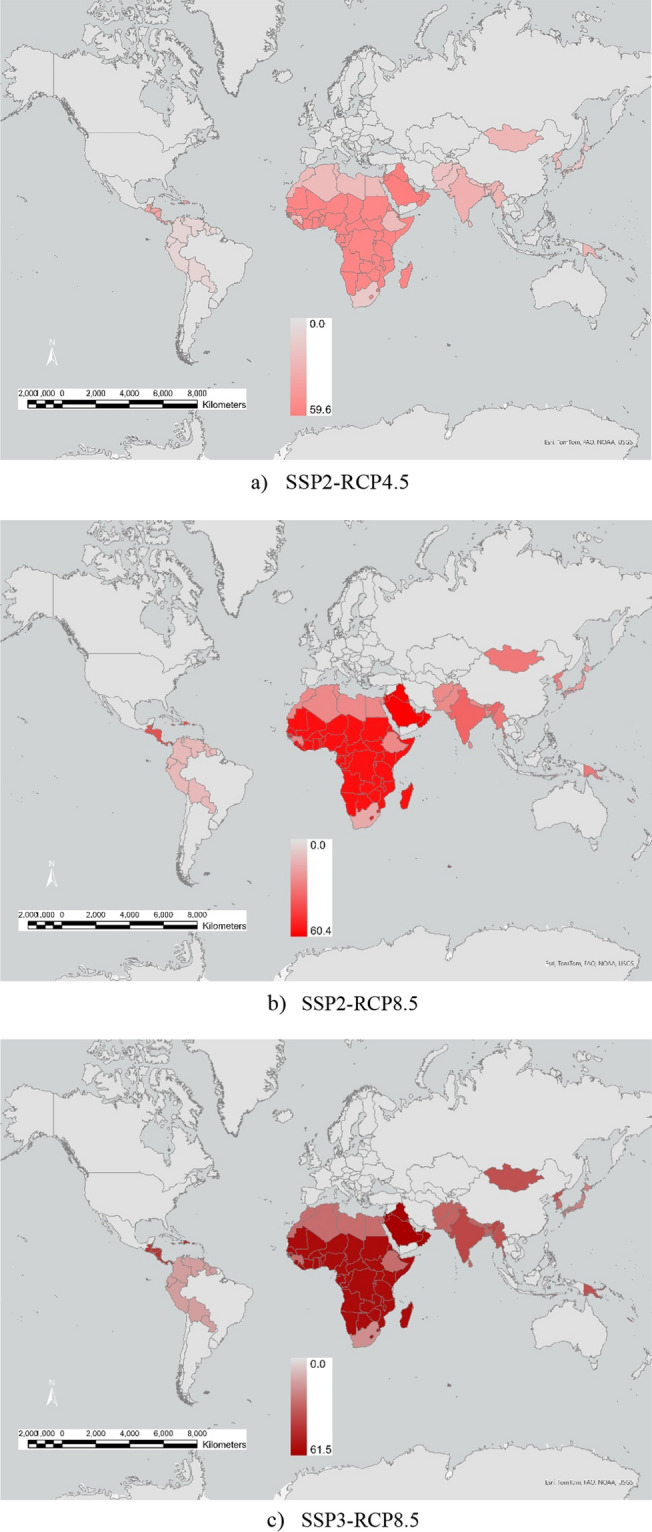
| Persons with severe food insecurity by region in 2050 relative to 2020 *(% population range)*. Model output; maps generated using ArcGIS Pro 3.3 (https://www.esri.com).

Heat stress and water stress from climate change both increase global food insecurity (see Fig. 2a–c). Overall, Africa is the most threatened in terms of severe food insecurity because of reductions in the continent’s food production due to water and heat stress and because of the projected increase in Africa’s population by 2050. Other regions with substantial increases in severe food insecurity include the Middle East, South Asia, and Central America.

For the SSP3-RCP8.5 case, domestic food production in many African countries will provide less than half of their domestic food demand (Fig. 2c). Some regions, such as China and ASEAN countries, switch from being net food exporters to food importers by 2050, with a need to import from food-producing regions that are impacted by climate change. Globally, the number of additional people with severe food insecurity by 2050, relative to 2020, for scenarios SSP2-RCP4.5, SSP2-RCP8.5, and SSP3-RCP8.5 are 556 million, 935 million and 1.36 billion, respectively.

In terms of prices and trade flows (i.e., exports and imports), the projected changes in our large dimensional model are complex, but the general results are robust. In all cases, model results show that there are substantial increases in food prices overall, and especially for the most extreme scenario and for those regions with high water stress. Our results show an increased flow of trade in agricultural commodities from low to high water stress countries and regions, given (in part) by the relative regional food price changes. Food exports to China, from lower water stress countries, increase across all three scenarios.

## Discussion

Our results highlight the regional and global magnitude of both heat stress and water stress on global food production. The value add of our modeling and projections are that we: (1) quantify both heat and water stress on global food production, noting that irrigation, in general, reduces the risks of water stress on crops relative to non-irrigated land^24^; (b) account for global commodity price changes (30 commodities) and the reallocation of resources across 30 countries/regions; (3) incorporate trade effects noting that climate change and water withdrawals, especially groundwater depletion, can transmit risks from water-stressed regions to regions without water stress via food trade^25^; and (4) allow producers and households to be forward-looking.

Climate change has already had a substantial and negative impact on global agricultural productivity, reducing a global measure of agricultural productivity by about 20% since 1970, with larger negative impacts in the Near East and North Africa^26^. Adverse impacts on productivity and yields will magnify with future climate change given longer duration, higher magnitude, and more frequent heat extremes^27^ and droughts^28,29^. To what extent technological change can offset yield declines from climate change is uncertain^30–33^. Future water availability for increased food production is also uncertain as the irrigated area in water-stressed regions is increasing, including in major food-producing regions such as China, India, Pakistan and the United State. In part, this is because the area in irrigation drives irrigated water withdrawals in these countries and because climate change will likely increase crop demand by further expanding irrigation^34–36^.

Global studies of the decline in terrestrial water storage^37,38^ show statistically significant declines in global storages over the period 1992–2020 in about half of all 1058 natural lakes and 922 global reservoirs. Over half of the decline in the storages is attributable to water withdrawals, increasing temperatures and potential evapotranspiration^4^. The OECD^7^ highlights that water stress, in the absence of effective policy actions in terms of water management, will significantly and negatively impact agricultural production in Northeast China, Northwest India and Southwest United States. These locations are in the world’s three largest food producing countries, all of which are currently net food exporters and have the biggest cumulative food footprints^39^. Other modeling in the existing literature highlight that the frequency of crop yield failures with climate change could be many times greater for key cereal crops (rice, soybean, maize, and wheat) that account for about two-thirds of food calorie consumption^40^ for China, India, and the USA over the period 2041–2060^41^.

A key limitation in our study is that results, using the WRI index, are based only on blue water (irrigation) sources. Water stress impacts on rainfed croplands are not yet available for use in our GTAP framework. Although we do include the impacts of heat stress on agriculture generally, it is important to recognize that rainfed croplands will not be impacted by heat stress only. Climate change alters precipitation patterns, thus modifying the spatial and temporal distribution of green water availability for both rainfed croplands and irrigated crops. While a shift from blue to green water crops can potentially and partially offset food production losses from heat and water stress in irrigated agriculture^42^, both blue water^43^ and green water^44^, globally, are under threat, especially in South Asia, East Asia, and the Middle East. Consequently, increases in agricultural production in either irrigated or rainfed agriculture in water-stressed regions will likely be at the expense of further groundwater depletion, inadequate stream flows and/or biodiversity loss. Agricultural extensification and increased rainfed cropping may pose additional sustainability challenges; over the period 2003–2019, about half of the 9% global increase in the cropland area removed natural vegetation and tree cover^45^.

Our results highlight the critical importance of quantifying the trade-offs in relation to water and food^46^ and climate change^47^. Food systems already contribute about one-third of anthropogenic greenhouse gas emissions^48^ and emissions from food production and consumption alone could contribute, under SSP2-RCP4.5, to almost a 1^°^C increase in the global surface temperature by 2100^49^. Food systems are also major contributors to biodiversity loss in Central and South America, and Africa^50^.

A major challenge is to increase regional and global food production without contributing to further climate change or increased water stress^51^, while ensuring sustainability^52,53^. This requires nothing less than a transformation in the world food systems^54–56^ simultaneous with much greater reductions in current greenhouse gas emissions from all sources, including agriculture.

## Methods

The GTAP-DynW model is a large dimensional CGE model that uses the extensive GTAP (Global Trade Analysis Project) Data Base Version 10^57^ in which countries or regions interact, importing goods and services from each other. GTAP-DynW is a (forward-looking) intertemporal rather than a recursive CGE model and includes 18 Agro-Economic Zones^58^ to characterize climate, soil, and terrain conditions pertinent to agricultural production^59^. Following Kompas and Van Ha^16^ and Kompas et al.^15^, the model also includes climate change damage functions.

In GTAP-DynW, within each country or region, a producer combines inputs (land, labor, capital, an intermediate good, and natural resources) to produce a single good or service, which is consumed domestically by regional households (i.e., final consumption) and producers (i.e., intermediate demand for products as inputs in the production of other commodities) or is exported to other international or regional households and producers. Producers account for future impacts and policy settings as per the following system of motion equations:

1 $${\dot{k}}_{r,t}={\varphi }_{r,t-}{\delta }_{r}{k}_{r,t}$$

2 $${\dot{\mu }}_{r,t}={\mu }_{r,t}\left[{{i}_{t}+\delta }_{r}\right]-\frac{{\phi }_{r}}{2}{\left(\frac{{\psi }_{r}}{{k}_{r,t}}\right)}^{2}{p}_{r,t}^{I}-{p}_{r,t}^{K}$$

where ${p}_{r,t}^{K}$ and *k*_*r,t*_ are the rental price of capital and the capital stock in region *r* at time *t*; ${p}_{r,t}^{I}$ is price of an investment good; *δ*_*r*_ is the capital depreciation rate; *ψ*_*r*_ is the capital increment from the (gross) investment activity; *i*_*t*_ is the global interest rate; ${\phi }_{r}$ is an investment increment coefficient; and *µ*_*r,t*_ is the shadow price of capital.

### Water stress effects

The production of agriculture output (*Q*_*j,t*_) is approximated by a constant elasticity substitution (*CES*) production function that includes the demand for commodity *i* for use by *j* (*QF*_*i,j,t*_) from both domestic and imported sources, and the value added in the industry *j* (*QVA*_*j,t*_). The demand of endowments (*QSE*_*i,j,t*_) that includes the 18 AEZ land use categories and natural resources for the value added in the industry *j* (*QVA*_*j,t*_) is given by:

3 $${QSE}_{i,j,t}=\left[\frac{{QVA}_{j,t}}{{afe}_{i,j,t}}\right]{\left({afe}_{i,j,t}\frac{{PVA}_{j,t}}{{PSE}_{i,j,t}}\right)}^{{\gamma }_{j,t}}$$

where *afe*_*i,j,t*_ is augmenting technological change of the endowments *i* by *j*; *PSE*_*i,j,t*_ is the market price of ‘sluggish’ endowment *i* (e.g., land which is hard to reallocate) used by industry *j*; *PV A*_*j,t*_ is the firm’s price of value added in industry *j*; and *γ*_*j,t*_ is the elasticity of transformation for sluggish primary factor endowments in the production of value-added in *j*.

Under the effect of water and heat stress, with climate change, the effectiveness of land use for agriculture or AEZ land decreases, where the relative reduction in the efficiency of land endowments is directly proportional to the relative increase in water stress. In GTAP-DynW, water stresses are derived from WRI^60^. This global GIS water data of 15,006 basins was spatially merged with the global GIS layers Esri-USGS^61^ to generate a geographical water stress projection for 174 countries, and then mapped to accord with GTAP-DynW’s 30 aggregate countries/regions. Water stress at a time by a region *i* is estimated from the projection of water stress in all basins *j* located in the region *i* with a weighted coefficient of *j* that is measured as the area or share of *j* in the total basin area in *i*, or

4 $$ws\left(i\right)={\sum }_{i=1}^{J}wsb\left(i,j\right)\frac{B\left(i,j\right)}{{\sum }_{i=1}^{J}B\left(i,j\right)}$$

where *wsb*(*i, j*) is the water stress of a basin *j* (in the set of *J* basins located in region *i*), *i* belongs to the GTAP-DynW’s 30 regions (*I*); and *B*(*i, j*) is the area of basin *j* located in *i*.

Water stress affects land use by AEZ by region and time in *QFE*_*i,j,t*_ and changes *QVA*_*j,t*_ and, thus, agricultural production (*Q*_*j,t*_). The water stress indicators are deviations (i.e., too much and too little) from the 2020 baseline under the effect of climate change, following WRI^60^, and are quantified only for irrigated agriculture. The magnitudes of the water stress shock by AEZ depends on the share of irrigated land in total land use in the region’s AEZ (*w*_*c,irr*_) and the change of irrigated water volume in that AEZ in a region (*dIW*_*c,t*_), is given by:

5 $$QSE_{i,j,t} = \mathop {\overbrace{{\frac{{L\left( {c,irr} \right)}}{{L\left( {c} \right)}}}}}\limits^{{w{_c,{irr}} }}{{dIW_{c,t} }}$$

where *c, j, t* represents 18 AEZ land types, agricultural commodity, and time.

To calibrate the impact of water stress on agricultural outputs, we assumed a constant elasticity of substitution (*CES*) production function and defined demands for intermediate inputs (*QF*_*i,j,t*_) by:

6 $${QF}_{i,j,t}=\left[{A}_{i,j,t}\frac{{Q}_{j,t}}{{afw2}_{j,t}{\left(\frac{{pf}_{i,j,t}}{{afw1}_{i,t}}\frac{1}{{ps}_{j,t}}\right)}^{{\gamma }_{j,t}}}\right]$$

where *Q*_*j,t*_ is the agricultural output of commodity *j*; *pf*_*i,j,t*_ is the firm’s price for input commodity *i* for use by *j*; *ps*_*j,t*_ is the supply price of commodity *j*; *A*_*i,j,t*_ is the composite regional variable of augmenting technology change; and *γ*_*j,t*_ is the elasticity of substitution among composite intermediate inputs in the agricultural sector *j*. Two specific augmenting technology change variables include a water stress factor (*afw*1_*i,t*_) for intermediate inputs and endowments used for production, and a region-specific average rate of intermediates augmenting technology change of *j* (*afw*2_*j,t*_).

The shock *afw*1_*c,j,t*_ depends on the weighted coefficient of irrigated land in total land use for an agricultural crop *w*_*irr,j*_, the change of irrigated water by time (*dIW*_*c,t*_), and the water stress coefficient to crop yields, or

7 $$dafw1_{c,j,t} = \mathop {\overbrace{{\frac{{L\left( {c,j,irr} \right)}}{{L\left( {c,j} \right)}}}}}\limits^{{w_{irr,j} }} \frac{1}{{dIW_{c,t} }}ws_{c,j,t}$$

where *c, j, t* represents 18 AEZ land types, agricultural commodity, and time. Water prices from water resources paid by water user industries are added to regional income, but also increase costs in these sectors that may cause a shift or reallocation of water use among water using industries. For a water price *pwater*(*t*) in a region *r*, the price index for purchases of *k* commodity by *j* sector in region *r* (*PFE*_*j,k,r,t*_) is given by:

8 $${PFE}_{k,j,r,t}=\left[{p}_{k,j,r,t}+{taxF}_{k,j,t}\right]+\left(\frac{{pwater}_{r,t}*{WIN}_{j,k,r,t}}{{VFA}_{k,j,r,t}}\right)$$

where *VFA*_*j,k,r,t*_ is the purchase and firm’s tax of *k* inputs for use by sector *j*; *p*_*j,k,r,t*_ is the market price of *k* to *j*; *taxF*_*j,k,r,t*_ is the tax on firm’s purchases of *k* by production *j*; *pwater*_*r,t*_ is water price at *t*; and *WIN*_*j,k,r,t*_ is the water intensity of *j* on *k*. Thus, a water stress shock causes a change in *PFE*_*j,k,r,t*_ and a shift in water withdrawals from domestic and imported sources depends on the domestic and import commodity mix.

### Heat stress effects

Damage functions provide the relationships between climate variables (such as average temperature, humidity, or extreme heat days) on productivity, income, and resource endowments^62^. Roson and Sartori^62^ provide the estimated parameters of damage functions for 120 GTAP countries and regions using GTAP9 with six climate impacts: sea level rise, variation in crop yields, heat stress effects on labor productivity, human health, tourism, and household energy demand. Projections from GTAP-DynW include damage functions related to heat stress and their impacts on agricultural outputs and labor productivity in the agricultural sector using GTAP10a. The heat stress shocks from global warming (e.g. losses in agricultural and labor productivity) are based on Kompas et al.^15^, Kompas and Van Ha^16^, and Roson and Sartori^62^.

### Food security effects

In GTAP-DynW, each food commodity contains nutritional components with different energy intake (calories). The aggregated nutritional supply in the region *r* (*S*(*r, t*)) (measured as Giga-calories (GCal)) is aggregated as a sum of nutritional supply from food production *i* or

9 $$S\left(r,t\right)={\sum }_{i=1}^{I}\frac{S\left(i,r,t\right)z\left(i\right)*1000}{{10}^{9}}$$

where *S*(*i, r, t*) is food production *i* (thousand tons); and *z*(*i*) is the nutrition conversion factors of food *i* for calculating that food’s energy content from one ton of food *i* to calories. The average daily nutrition intake *a* required for human food security is taken as given, and varies by country and region (source data: FAO^22,63,64^) and is given by:

10 $$F\left(r,t\right)=\frac{S\left(r,t\right)*{10}^{9}}{a*365*{10}^{6}}$$

where *a* is average daily nutrition in calories. Global food production (GCal) is the sum of all regional food supply and the total population across regions is *F(r,t)*.

The number of persons with severe food insecurity, in millions, is determined by the gap in the minimum calorie demand and the available production and is given by:

11 $$IF\left( {r,t} \right) = \mathop {\frac{{\overbrace{{\left[ {S(r,0) - S(r,t)} \right]}}10^{9} }}{{a*365*10^{6} }}}\limits^{{ds\left( {r,t} \right)}}$$

where *dS*(*r, t*) is the reduction of food production in region *r* at *t* to the base nutritional supply (*S*(*r,* 0)).

The food insecurity rate (*RIF*(*r, t*)) is the ratio of the number of persons with severe food insecurity over the total population of that country or *POP* (*r, t*):

12 $$RIF\left(r,t\right)=\frac{IF\left(r,t\right)}{POP\left(r,t\right)}$$

The global reduction of food production (*dS*(*t*)) is the sum of the reduction of food supply (*dS*(*r, t*)) across all regions. The global number of persons with severe food insecurity (*IF* (*t*)) is the sum of all regions’ persons with severe food insecurity resulting from the reduction of food supply (or *IF* (*r, t*)).

### Supplementary Information

Supplementary Information.

## Data Availability

Model code and data sources available at: 10.5281/zenodo.8248417
