# Supplementary material for: Global impacts of heat and water stress on food production and severe food insecurity
Source: Sci Rep. 2024 Jun 22;14:14398. doi: 10.1038/s41598-024-65274-z (PMC11193756; doi:10.1038/s41598-024-65274-z)
Supplement: Supplementary file 1 — Supplementary Information. [file 41598_2024_65274_MOESM1_ESM.pdf]

Supplementary Information

Supplementary Information for the 'Global impacts of heat and water stress on food production and severe food security' manuscript.

1 GTAP-DynW Model Structure

Fgure S1 illustrates the computational approach and model structure in GTAP-DynW.

Figure S1: GTAP-DynW

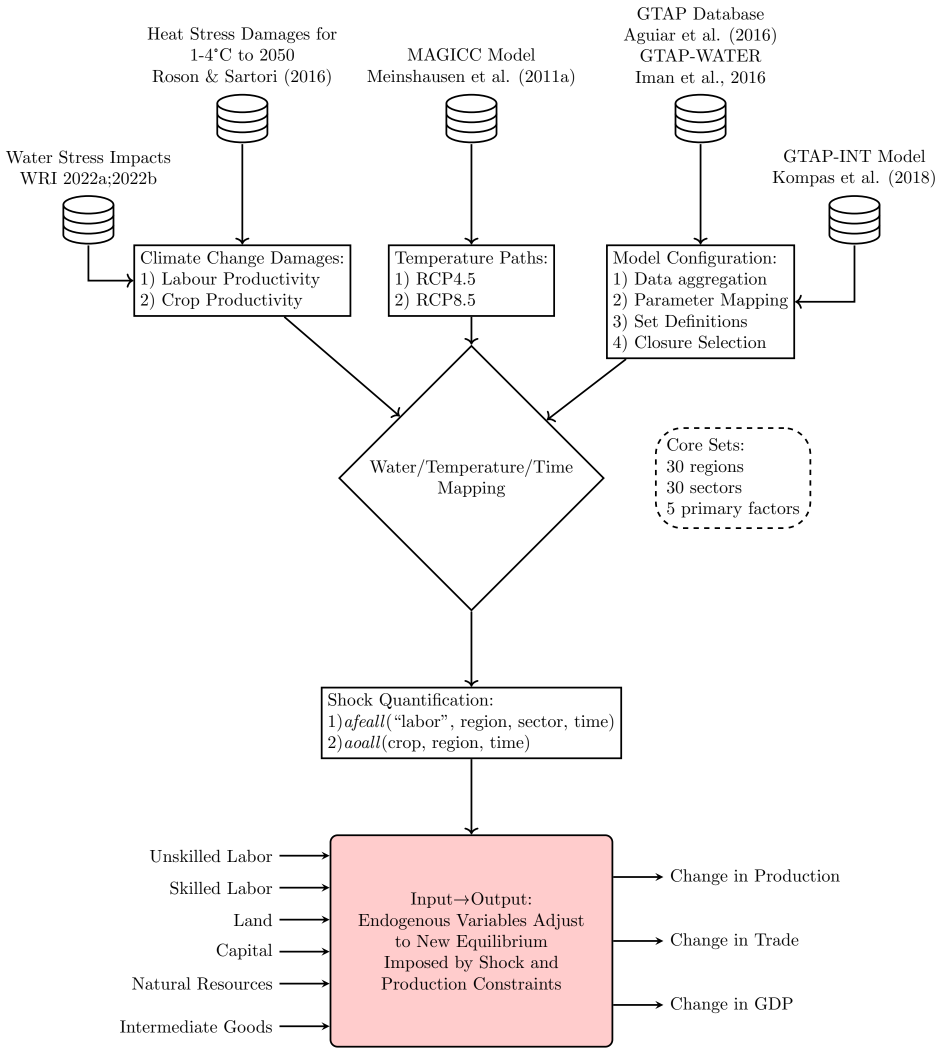

Note: GTAP-DynW is a large dimensionbal CGE model with heat stress and water stress components, temperature pathways, GTAP databases and a core GTAP-INT computational model. Primary factors input factors are as indicated and labour is augmented over time through population growth dependng on assumed SSP. Shocks to labour aand agricultural productivity are channelled through heat and water stress damage functions, by input, region, sector and time, altering regional output and global trade patterns. Addtional data sources are as indicated below in the Supplementary Information.

2 Agro-Ecological Zones

Global map of the 18 AEZs used in GTAP-DynW is provided in Figure S2. A precise mapping of AEZs to regions in GTAP-DynW is given in Table S1-S2.

Figure S2: Global Agro-ecological Zones (AEZs)

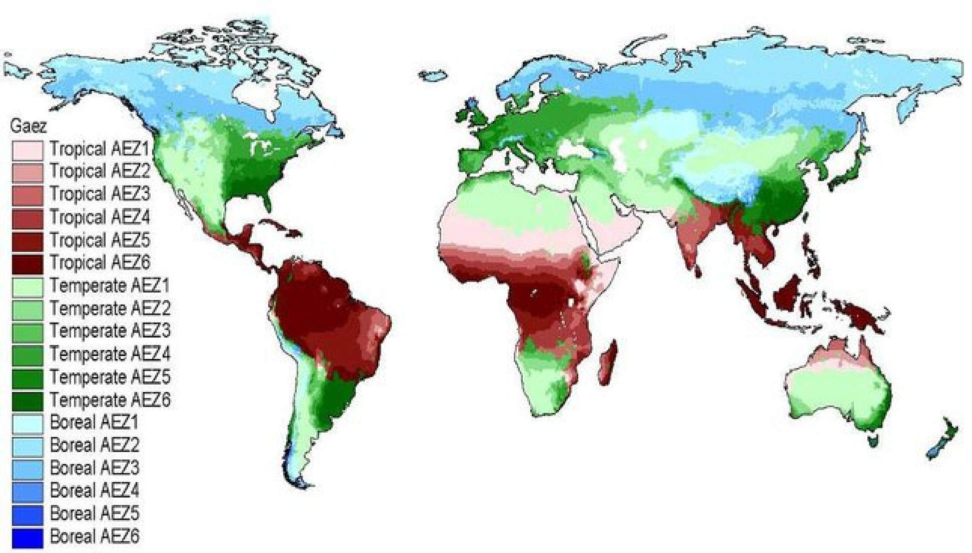

Source: [19].
Note: A global map by agro-ecological zones (AEZs 1-18) and regions used in the GTAP- BIO model is provided in Lee (2004). 1-18 AEZ in GTAP-DynW are in order by Tropical AEZ1-6; Temperate AEZ1-6; and Boreal AEZ 1-6. Mapping of AEZs to regions in GTAP-DynW are indicated in Table 1.

Table S1: Baseline Irrigated Water Circulation by AEZ1-9 and Region in GTAP-DynW *(Mil m3/year)*

	Region	**AEZ1-9**								
		aez1	aez2	aez3	aez4	aez5	aez6	aez7	aez8	aez9
1	aus	9.6	37.3	756.4	66.0	0.2	0.0	1,354.2	685.6	1,130.1
2	bra	-	167.2	86.8	553.9	622.4	156.1	-	-	-
3	caf	5,214.7	890.3	570.7	319.0	111.2	1,538.7	645.1	17.8	160.5
4	cam	-	-	68.9	727.3	842.8	325.3	-	-	-
5	can	-	-	-	-	-	-	-	36.7	2.0
6	ceu	-	-	-	-	-	-	-	-	-
7	chn	-	-	-	113.8	498.5	491.9	133,711.9	62,945.8	112,653.4
8	deu	-	-	-	-	-	-	-	-	150.2
9	eao	-	-	-	-	-	-	0.2	4.0	157.2
10	eew	-	-	-	-	-	-	37,059.0	52,014.7	2,364.5
11	fra	-	-	-	-	-	-	-	38.5	40.3
12	gbr	-	-	-	-	-	-	-	-	-
13	ind	64.8	16,317.8	86,362.5	44,476.9	707.1	-	19,065.3	86,927.3	107,941.4
14	ita	-	-	-	-	-	-	-	46.1	28.7
15	jpn	-	-	-	-	-	-	-	-	173.0
16	kor	-	-	-	-	-	-	-	-	-
17	mex	10,689.9	2,859.0	5,535.1	1,218.5	389.9	84.3	8,301.1	3,557.1	7,092.8
18	naf	587.8	102.5	53.5	17.4	2.3	264.8	4,543.7	83.1	269.8
19	nsa	6,224.8	878.3	2,164.9	2,158.0	3,415.2	1,044.7	2,343.7	1,673.5	396.2
20	nzl	-	-	-	-	-	-	-	0.0	25.3
21	osa	585.3	231.9	245.9	95.3	6.6	197.6	470.4	40.9	130.9

Table S1 – *Continued from previous page*

	Region	AEZ1-9								
		aez1	aez2	aez3	aez4	aez5	aez6	aez7	aez8	aez9
22	rus	-	-	-	-	-	-	-	511.0	165.1
23	sas	24,604.0	-	263.2	6,095.4	7,466.4	1,142.1	398,746.8	24,088.1	20,618.7
24	ssa	-	-	156.5	63.3	34.7	11.6	9,996.3	5,138.7	1,239.5
25	tur	-	-	-	-	-	-	66.8	7,937.9	1,870.8
26	usa	-	-	-	-	-	-	21,692.3	21,549.6	7,897.6
27	weu	-	-	-	-	-	-	-	72.2	655.2
28	zaf	44.1	18.0	5.3	0.4	0.1	-	538.8	147.7	207.6
29	asean	-	-	-	24,761.9	26,677.6	58,641.2	-	-	-
30	me	331,627.0	-	-	-	-	-	332,770.1	60,985.3	10,242.2
	Sum	379,652.0	21,502.2	96,269.6	80,667.3	40,775.1	63,898.2	971,305.7	328,501.8	275,612.8

Note: Authors' calculation of baseline irrigated water based on [14] and [2]. See Table S3 for relevant country and region codes.

Table S2: Baseline Irrigated Water Circulation by AEZ10-18 and Region in GTAP-DynW *(Mil m3/year)*

	Region	AEZ10-18								
		aez10	aez11	aez12	aez13	aez14	aez15	aez16	aez17	aez18
1	aus	433.6	702.7	131.4	-	-	0.0	1.3	-	-
2	bra	0.1	2.9	1,213.9	-	-	-	-	-	-
3	caf	214.3	150.2	212.2	-	-	-	-	-	-
4	cam	84.1	22.9	2.0	-	-	-	-	-	-
5	can	5.0	17.1	-	114.4	23.2	1.5	0.1	-	-
6	ceu	709.6	-	-	-	0.0	0.3	0.0	-	-
7	chn	19,649.0	17,908.9	48,444.0	1,734.7	90.4	280.9	0.1	-	-
8	deu	205.6	114.2	-	-	-	0.0	-	-	-
9	eao	1,759.4	1,133.8	-	0.8	6.8	111.4	-	-	-
10	eew	694.8	16.2	1.7	27.4	1,399.1	6.8	0.1	-	-
11	fra	196.9	568.8	34.4	0.0	0.0	0.0	-	-	-
12	gbr	88.7	257.9	7.8	-	-	0.0	0.0	-	-
13	ind	9,320.8	973.8	1,176.5	-	-	-	-	-	-
14	ita	95.2	421.1	4.4	0.0	0.0	0.0	-	-	-
15	jpn	6,743.0	16,793.9	9,084.4	-	-	172.5	-	-	-
16	kor	531.9	835.6	-	-	-	-	-	-	-
17	mex	1,087.3	57.8	2.3	-	-	-	-	-	-
18	naf	380.2	147.9	901.4	-	-	-	-	-	-
19	nsa	408.7	44.5	193.8	-	-	-	-	-	-
20	nzl	69.1	247.9	623.4	-	-	0.2	11.9	-	-
21	osa	76.9	61.1	48.8	-	-	-	-	-	-
22	rus	72.9	0.4	-	26.6	27.7	33.0	-	-	-
23	sas	22,362.6	1,396.8	3,863.9	0.7	-	-	-	-	-
24	ssa	1,892.3	121.3	405.8	90.5	740.4	192.6	31.1	-	-
25	tur	158.0	7.6	0.1	0.0	24.3	1.9	-	-	-
26	usa	1,077.0	1,063.5	1,916.2	189.0	185.4	2.0	0.0	-	-
27	weu	263.1	331.6	25.3	0.1	4.6	0.7	0.0	-	-
28	zaf	121.8	41.3	3.8	-	-	-	-	-	-
29	asean	0.2	19.3	190.3	-	-	-	-	-	-
30	me	1,255.4	-	-	-	-	-	-	-	-
	Sum	69,957.4	43,461.0	68,487.9	2,184.3	2,502.0	803.9	44.7	-	-

Note: Authors' calculation of baseline irrigated water based on [14] and [2]. See Table S3 for relevant country and region codes.

3 Data

The GTAP data for GTAP-DynW is the GTAP-AEZ database Version 10a [12], which is calibrated for the world economy across 65 (tradeable) commodity sectors for 141 countries/regions (with the base year 2014). These 141 countries/regions account for 98% of world GDP and 92% of the world's population [1]. In GTAP-DynW, the countries/regions and commodity sectors are aggregated into 30 countries/regions and 30 sectors (see Tables S3 and S4 for details). The GTAP-DynW sluggish (i.e., relatively unmoveable) land endowment is disaggregated into 18 categories by [1]. It is important to note that countries listed within a given region have different water and heat stress baselines.

The supporting database of agricultural production and land in each GTAP-DynW region is drawn from [8]. Thirteen agricultural sectors are analyzed, including: paddy; wheat; cereal; vegetables ; oilseed; sugar can; fibers; other crops; livestock; poultry; meat and animal products; dairy, and wool[1]; oilseed; sugar can; fibres; other crops; Livestock; pountry; meat and animal products; dairy, and wool.[2]

We incorporate GTAP-DynW's data for AEZ regions and industries with the irrigation water data from GTAP-Water data (Version 9) [12] as analyzed by [14]. GTAP-DynW's database represents cropping activities in eight distinct sectors: paddy rice, wheat, coarse grains, vegetable and fruits, oilseed, sugar crops, plant-based fiber, and other crops. AEZ land use is decomposed into irrigated and rain-fed harvested area from [14] for these eight distinct sectors. Overall, irrigated yield is higher than rain-fed yield (Iman et al., 2016). The share of irrigated area in total cropland by AEZ, region, and agricultural industries is based on [14]. GTAP-DynW's 30 regions concord with aggregated areas from [14].

Geographical data sources for global aqueduct water by basins and countries are drawn from [26] and analyzed in [10]. Projections of water stress by climate change scenario are from [25] and [20]. Other global Geographic Information System (GIS) spatial data are from [4].

We also apply the parameter of water stress impacts on wheat for RCP4.5 and RCP8.5 from moderate and severe water stress levels in [27] with consideration from [21] and [11]. The water stress impacts for other crops are drawn from [23]. The base data of food supply [7], and the nutritional content of foods and grains are from [6] and [5]. The average yearly dietary consumption per capita is from [5], by country/region. Heat stress indexes are again drawn from [17], [18] and [22].

4 WRI Projections of Global Water Stress

The two key recent and most relevant studies of water stress by climate change scenario, particularly in irrigated areas, are [9] and [25]. Within a coherent AEZ framework, [9] provides a new methodology for estimating irrigation water requirements under current and future changes by climate and socioeconomic conditions to 2080. In that study, [9] projects global and re-

[1]The list of vegetables products includes cabbages and other brassicas, artichokes, asparagus, lettuce and chicory, spinach, tomatoes, cauliflowers and broccoli, pumpkins, squash and gourds, cucumbers and gherkins, eggplants (aubergines), chilies and peppers, green, onions, shallots, green, onions, dry, garlic, leeks, other alliaceous vegetables, beans, green, peas, green, vegetables, string beans, carrots and turnips, okra, mushrooms and truffles, vegetables, bananas, plantains and others, oranges, tangerines, mandarins, clementines, satsumas, lemons and limes, grapefruit, fruit, citrus, apples, pears, quinces, apricots, cherries, peaches and nectarines, plums and sloes, stone fruit, strawberries, raspberries, currants, blueberries, berries, grapes, watermelons, melons, other, figs, mangoes, mangosteens, guavas, avocados, pineapples, dates, persimmons, kiwi fruit, papayas, tropical fresh fruit, hops, pepper, chilies and dry peppers [8].

[2]Livestock (in thousand heads) includes buffaloes, cattle, goats, horses, pigs and sheep. Poultry sectors (in million heads) include chickens, ducks, and turkeys.

gional agricultural water demand for irrigation using a new socioeconomic scenario developed by IIASA, with and without climate change. Water deficits of crops are projected in the FAO-IIASA-AEZ model, which is based on daily water balances at 0.5^o latitude x 0.5^o longitude and then aggregated to regions and the globe. While the study by [9] is valuable for projecting water deficit under the effect of climate change, it is limited to 13 regions (with different regional concordances to GTAP).

In GTAP-DynW, the shock for water stress is instead based on [25], which is extracted from the Geographic Information System (GIS) WRI layers for future water demand, availability, and water stress for RCP4.5-SSP2, RCP8.5-SSP2, and RCP8.5-SSP3, covering 15,006 basins for the decadal ranges of the 2020s, 2030s, and 2040s (see [20]). SSPs are used for relative population projections only. We also consider alternative layers for future global water stress and current water data for 25,010 basins [26], and other geographical features from [4]. Other GIS layers of water and country characteristics are drawn from [26] and [4].

The WRI water stress projections show the impacts of climate change from global warming in clear terms, with water stress increasing by both the level of water stress and the number of basins moving to higher water stress categories. Using [25], we constructed Figure S3 to represent the water stress indexes for the baseline (25,008 basins) and projections of global basins for RCP4.5-SSP2, RCP8.5-SSP2, and RCP8.5-SSP3. [25] classified five water stress categories (presented as horizontal lines in Figure S3), including <1: Low water stress; 1-2: Low to Medium water stress; 2-3: Medium to High water stress; 3-4: High water stress; >4: Extremely High water stress. The dark red line represents the case of water stress greater than 5 (or the top limit for the baseline). Following [20] and [25], overall future water stress would increase, given climate change, especially so across a large number of regions, including the Mediterranean, the Middle East, the North American West, Eastern Australia, West Asia, Northern China, and Chile.

Figure S3: Projection of Water Stress of Global Basins by Climate Change Scenario

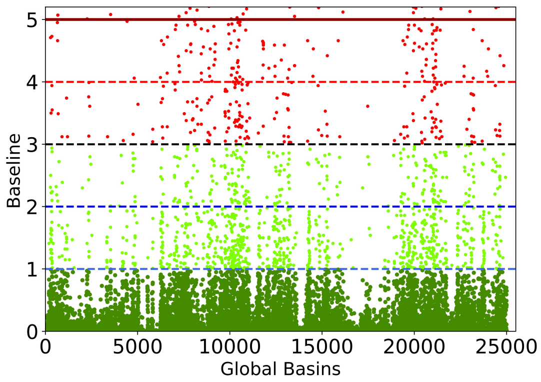

i) Baseline water stress

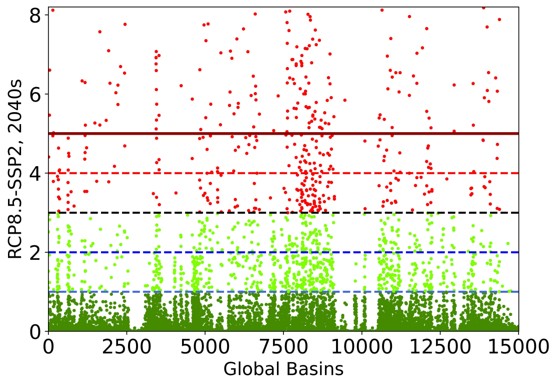

ii) The projection for the 2040s of RCP4.5-SSP2

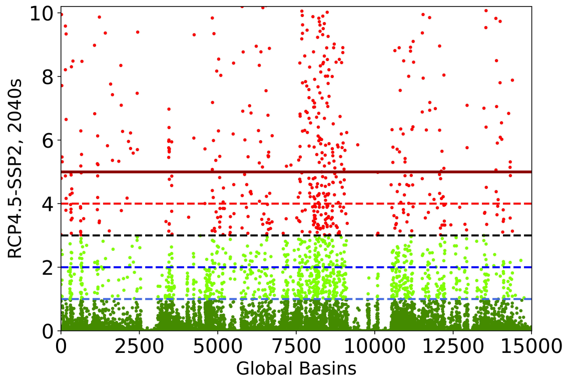

iii) The projection for the 2040s of RCP8.5-SSP2

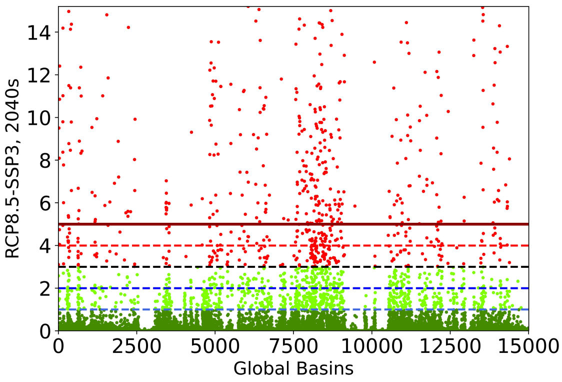

iv) The projection for the 2040s of RCP8.5-SSP3

Source data: Extracted from the GIS spatial layers of [25]. Note: Water Stress: <1: Low Water Stress; 1-2: Low to Medium Water Stress; 2-3: Medium to High Water Stress; 3-4: High Water Stress; >4: Extremely High Water Stress. The solid or dark red line indicates water stress > 5, the top of the initially indicated water stress limit. Red dots indicate high to extreme high water stress. Note the change in vertical scale in Figures (i) to (iv), with the index moving to as high as 14, with a substantial increase in the frequency or number of water basins > 3 or in the High Water Stress range.

Table S3: GTAP-DynW Regions

No	Region	Countries included
		**North America**
1	USA	United States
2	CAN	Canada
3	MEX	Mexico, Rest of North America
		**South & Central America**
4	BRA	Brazil
5	CAM	**Central South America:** Costa Rica, Guatemala, Honduras, Nicaragua, Panama, El Salvador, Rest of Central South America, Dominica, Jamaica, Puerto Rico, Trinidad and Tobago
6	NSA	**Northern South America:** Bolivia, Colombia, Ecuador, Paraguay, Peru, Venezuela, Rest of South America
7	SSA	**Southern South America:** Argentina, Chile, Uruguay
		**Europe & Eurasia**
8	CEU	**Central Europe:** Czech Republic, Estonia, Hungary, Latvia, Lithuania, Poland, Slovakia, Slovenia, Bulgaria, Croatia, Romania
9	DEU	Germany
10	EEW	**East Europe & West Asia:** Albania, Belarus, Ukraine, Rest of Eastern Europe, Kazakhstan, Kyrgyztan, Tajikistan, Rest of Former Soviet Union, Armenia, Azerbaijan, Georgia
11	FRA	France
12	GBR	United Kingdom
13	ITA	Italy
14	TUR	Turkey
15	RUS	Russia
16	WEU	**Other Western Europe:** Austria, Belgium, Denmark, Finland, Greece, Iceland, Ireland, Luxembourg, Netherlands, Norway, Portugal, Spain, Malta, Sweden, Switzerland, Rest of Western Europe
		**Middle East**
17	ME	Bahrain, Iran, Israel, Jordan, Kuwait, Oman, Qatar, Saudi Arabia, UAE
		**Africa**
18	CAF	**Central Africa:** Benin, Burkina Faso, Cameroon, Cote d'Ivoire, Ghana, Guinea, Nigeria, Senegal, Togo, Rest of Western Africa, Central Africa, South Central Africa, Kenya, Rwanda, Tanzania, Uganda, Rest of Eastern Africa
19	NAF	**North Africa:** Egypt, Morocco, Tunisia, Rest of North Africa, Ethiopia
20	OSA	**Other Africa:** Madagascar, Malawi, Mauritius, Mozambique, Zambia, Zimbabwe, Botswana, Namibia, Rest of Africa
21	ZAF	South Africa
		**Asia-Pacific**
22	ASEAN	Brunei, Cambodia, Lao, Malaysia, Philippines, Singapore, Thailand, Viet Nam, Rest
23	AUS	Australia
24	CHN	China & Hong Kong
25	IND	India
26	JPN	Japan
27	KOR	Korea
28	NZL	New Zealand
29	SAS	**South Asia:** Bangladesh, Nepal, Pakistan, Sri Lanka, Rest of South Asia
30	EAO	Rest of Oceania, Mongolia, Taiwan, Rest of East Asia

Table S4: GTAP-DynW Sectors

No	Codes	Model Sectors	GTAP V10 Sectors
1	pdr	Paddy rice	Paddy rice
2	wht	Wheat	Wheat
3	gro	Cereal grains nec	Cereal grains nec
4	ocr	Plantation	Vegetables, fruit, nuts; Oil seeds; Sugar cane, sugar beet; Plant-based fibres; Crops nec

Table S4 – continued from previous page

No	Codes	Model Sectors	GTAP V10 Sectors
5	ctl	Livestock	Cattle, sheep, goats,horses
6	oap	Animal products nec	Animal products nec
7	frs	Forestry, fishing	Forestry, fishing
8	coa	Coal	Coal
9	oil	Oil	Oil
10	gas	Gas	Gas
11	omn	Minerals nec	Minerals nec
12	omt	Livestock Products	Wool, silk-worm cocoons; Meat: cattle, sheep, goats, horse; Meat products nec
13	mil	Dairy	Dairy products, raw milk
14	ofd	Food processing	Processed rice; Sugar; Food products nec; Beverages and tobacco products, Vegetable oils and fats
15	tex	Textiles, wear	Textiles; Wearing apparel; Leather products
16	lum	Wood and paper	Wood products, Paper products, publishing
17	p_c	Petroleum, coal products	Petroleum, coal products
18	crp	Chemical, rubber, plastic prods	Chemical, rubber, plastic prods
19	nmm	Mineral products nec	Mineral products nec
20	i_s	Ferrous metals, Metals nec, Metal products	Ferrous metals, Metals nec, Metal products
21	omf	Manufacturing	Motor vehicles and parts; Transport equipment nec; Electronic equipment; Machinery and equipment nec; Manufactures nec
22	ely	Electricity	Electricity
23	gdt	Utilities	Gas manufacture, distribution; Water
24	cns	Construction	Construction
25	otp	Transport nec	Transport nec
26	wtp	Sea transport	Sea transport
27	atp	Air transport	Air transport
28	obs	Services	Communication, Communication; Trade; Financial services nec; Insurance; Business services nec; PubAdmin/Defence/Health/Educat
29	ros	Recreation	Recreation and other services
30	dwe	Dwellings	Dwellings

5 Shock Variables for Climate Change Scenarios

Table S5 presents Data Sets in the model and the shock variables for water stress impacts in this study, including shock on the quantity of AEZ land use and shock of water stress on agricultural production.

Table S5: Data Sets and Shock Variables

	Variables	Contents	
		**Sets**	Size
1	i=TRADE_COMM	Traded commodities	30
2	r=REG	Region	30
3	j=FIRM_COMM	Commodities demanded by firms	40
4	b=PROD_COMM	Produced commodities	31
5	c=ENDW_COMM	Endowment commodities	22
6	d= ENDWS_COMM	Slughish endowments (18 AEZ lands & natural resources)	19
7	e= ENDWL_COMM	Land Endowments (AEZ1-18)	18
8	t=alltime	time 2022-2100	79
		**Selected Intertemporal Shocks for Water Stress's Impacts (%)**	Average/Region
		(i) Effect on Land Use with Irrigated Water	

Table S5 – continued from previous page

	Variables	Contents	
3	*dQSEc,t,r*	Shock on quantity of AEZ land use in region r (%/year)	-0.1 to -3.1
		(ii) Shock of Water Stress on Agricultural Production (*%/year*)	
9	*dafw1j,t,r*	Shock of water stress on agricultural production in region r	0.1 to 5.4

6 Water Supply and Water Availability

Following [20], the WRI projections were developed primarily by general circulation models from the Coupled Model Inter-comparison Project-Global Circulation Models (CMIP5-GCMs) [24], and SSP scenarios from [13]. The WRI projections provide for 15,006 global basins in the form of GIS spatial layers, including water withdrawal and consumptive use (demand), water supply, water stress, and intra-annual (seasonal) variability for the 2020s, 2030s, and 2040s by RCP4.5 and RCP8.5-SSP2 and RCP8.5-SSP3.

[25] estimates water supply at basin level (for 15,006 basins) from runoff values extracted from an ensemble of CMIP5-GCMs, which provides valuable insights about the climate system and the processes responsible for climate change and variability. More than 20 modeling modules are performing for the 50 CMIP5 model simulations [24]. An essential input of CMIP is from the Global Coupled Ocean-Atmosphere General Circulation Models (coupled GCMs), which detect both anthropogenic effects over the past century and project future climate changes due to human activities and energy fuel-mix changes. CMIP has archived output from both constant forcings ('control run') and perturbed (1% per year increasing atmospheric carbon dioxide) simulations using summarized results from 18 CMIP models [3]. Representing a broad lineage of models from geographically and diverse modeling approaches, six Global Circulation Models (GCMs) were selected to reproduce the mean and standard deviation of historical runoff using macro variables from the RCP4.5 and RCP8.5 scenarios. Several GCMs had results for multiple ensemble members across the two climate scenarios (13 for RCP4.5 and 17 for RCP8.5). In particular, [25] fit generalized extreme value (GEV) distributions separately for each pixel over the historical period data (1950–2005) for each GCM run and the corresponding Global Land Data Assimilation System (GLDAS-2) data. The GCM values are corrected by matching distributions.

The WRI water supply indicator is total blue water (renewable surface water), which projected change to be equal to the 21-year mean around the target year divided by the baseline period. [20] estimate total blue water (Bt) and available blue water (Ba) from bias-corrected runoff values, which were resampled to 1 km x 1 km spatial layers and summed into hydrological catchments for the downstream water flow-accumulation in rivers. Following [10], [25] used an approach of sparse catchment- to-catchment flow accumulation to estimate water supply to a catchment.

7 Water Withdrawals and Consumption

Water withdrawals and consumption for agriculture, industry, and domestic users were projected from historical data and macro-outlooks of GDP, population, and urbanization. The variables employed for water demand projection include area equipped for irrigation; agricultural land area (including both irrigated and rain-fed agriculture); irrigation efficiency; industrial water withdrawals; domestic water withdrawals; GDP per capita; urbanization; baseline water stress, population density; and world population. While most macro variables are from FAO and World

Bank, the baseline water stress for 25,008 global basins are from [26] and [10]. [25] measures water demand as water withdrawals, with the projected change in water withdrawals equal to the summarized withdrawals for the target year, divided by the baseline year.

8 Irrigation Withdrawals

Agricultural irrigation (by far the most significant withdrawals) is unique among water users because its withdrawals depend strongly on climate (as evaporative demand) and the extent and efficiency of irrigation.

In agriculture, the projected change in water withdrawals (Uag) equals the summarised withdrawals for the target year, divided by the baseline year, 2010. Since water consumptive irrigation use (Cag) varies based on climate, [25] estimate Uag and Cag for each year. First, [25] projected country-level irrigated area, then spatially distributed the irrigated area within each country, and used climate projections to estimate the consumptive use over the projected irrigated area. [25] explicitly projected changes in the spatial extent of irrigation to incorporate the effect of climate over-irrigation areas. The projections of the irrigated area by country were estimated using mixed effects regression of space equipped for irrigation ($AEI_{i,t}$) from [8] for a country at the year as a function of the socioeconomic variables. To prevent projections from exceeding available agricultural land, the response variable was modelled as the logit-transformed proportion of agricultural land equipped for irrigation in total agricultural land for the country in year t. [20] employs a fit coefficient to predictor variables, including country-specific intercepts (with specific features of policies, geographical climate conditions, etc., and world population and international agricultural trade). [20] converted the regression model's projections to the irrigated area by using the predicted proportion (using the inverse-logit function techniques), multiplying by the area irrigated, and finally multiplying by the ratio of the area irrigated to the area equipped for irrigation.

The projection of irrigated area at the country level was distributed spatially within countries to pixels based on the likelihood of irrigation expansion (LIE) dataset. For each country and scenario, [25] projects the change in irrigation area (as the difference between the projected area for the target year and the baseline year). [25] generated one estimate of the extent of irrigated area for each of the target decades/years, the 2020s, 2030s, and 2040s.

9 Irrigation Consumption

Irrigation consumption was estimated following the FAO methodology of consumptive irrigation use (ICU), excluding crop-specific evapo-transpiration factors. ICU is the annual depth of water needed to fulfill the deficit between crop consumption with ample water and crop consumption with rainfed conditions. [25] calculated ICU as potential minus actual evapo-transpiration.

The irrigation water requirement (IWR) is the water required for optimal crop growth, including consumptive and non-consumptive purposes. The water requirement ratio (WRR), or irrigation efficiency, is the water required by crops to meet their evapo-transpiration needs divided by the amount of water withdrawn. This ratio is less than one because of water leakage or other losses in the irrigation system.

10 Water Stress

Following [10], the water stress at time t (WS_t) is estimated as the ratio of water withdrawals (UW_t) to available blue water (Ba_t) on an average annual basis

$$WS_t = \frac{UW_t}{Ba_{[t-10:t+10]}} \tag{1}$$

Available blue water Ba is flow-accumulated runoff minus upstream consumptive use over catchments. [25] computed Ba as the mean of the 21 years around the projected year. The baseline is the average value of the 1950–2010 period.

11 Heat Stress

Heat stress, determined by high temperature and humidity, causes more frequent pauses, work interruptions, risk of injury and lower labor productivity generally. The effectiveness of any adaptation measures (such as acclimatization and air conditioning) can be limited and dependent on the context [22]. This is especially the case for outside work in agricultural regions. The study by [16] applied physiological evidence regarding the effects of heat, climate guidelines for safe work environments, climate modeling, and global distributions of working populations to estimate the impact of heat stress on future labor productivity. Based on [16], [22] estimated a heat damage function, which is the relationship between average temperature and labor productivity. In particular they, estimated a "wet bulb globe temperature" (WBGT), which is applied in most standards working environments to protect workers from heat injury. We rely on this work to claibrate GTAP-DynW.

The estimates for the average monthly WBGT provided by [22] are drawn from the following:

$$WBGT = 3.94 + 0.567T + 0.393E \tag{2a}$$

with

$$E = (RH/100) * 6.105 * e^{[17.27T/(237.7+T)]} \tag{2b}$$

$$RH = 67.1082 - 0.8438T + 0.2305P - 0.0005P^2 \tag{2c}$$

where T is the average air temperature in ^{0}C; E is the average absolute humidity in hPa; RH is the average relative humidity in %; and P is the precipitation (in millimetres). Equation (2) includes (i) the WBGT function woith respect to T and E, equation (2)a; the humidity function with respect to T and RH, or equation (2)b; and the relative humidity function to T and P, equation (2)c.

The study by [16] produced a graph of 'work ability' as the maximum percentage of an hour a worker should be engaged in labour, covering four different work intensities by temperature. Based on this, [22] computed the % level of productivity for all months, sectors, and countries. Monthly values are subsequently aggregated to a yearly average since time steps in GTAP are annual, scaling up temperature levels from 1 to 5 ^{0}C, assuming the monthly temperature distribution will be unaffected and relative humidity stays the same. Finally, they computed the relative % change in (annual) productivity with respect to the baseline for 140 countries and regions, over three sectors: Agriculture (A), Manufacturing (M), and Services (S), and for 1-5^{0}C increases in average temperature, bringing about a total of 140 x 3 x 5 = 2100 estimated parameter values.

In this study, the shocks of heat stress on labor productivity are dawn directly from [22] by two climate change scenarios (RCP4.5 and RCP8.5). In 2090, global average warming levels would be 1.7^{0}C to 3.2^{0}C for RCP4.5 and 3.2^{0}C to 5.4^{0}C for RCP.85, respectively [15]. GTAP model use of this approach can also be found in [17].

12 Model Limitations

The impacts of climate change on water resources and its impact on agriculture are certainly complex, varying by region, industry, and time. Our shocks in GTAP-DynW are based on currently available and up-to-date data, parameters, and possible global warming projections. These may change over time and the simulation model will have to be adjusted. There are three addtional limitations that also need further research.

First, the amount of water by AEZ and regions is estimated from water intensity per hectare by regions, which is derived from AEZ areas and water use by AEZ from [14]. Given the disparity between the 19 regions by [14] and 30 regions in GTAP-DynW, there is a deviation in baseline water estimates by AEZ in the 30 regions of GTAP-DynW. We have tried to account for this but improving the AEZ water database with more accurate data by regions and agricultural sectors is needed. Second, the parameters for water stress impacts on agricultural production vary by regions and farming industries. The precise water stress impact on each agricultural commodity needs further work by crop and specific agricultural output. We include all that is currently available but more precise and fully articulated impacts would be invaluable. Finally, GTAP-DynW covers a large basket of agricultural commodities aggregated into 14 different types, assumed to be a 'basket' of food for nutrition. That is a common procedure. However, some other local food sectors and types (such as in Asia and Africa) are also influenced by water stress but are not available in GTAP or FAO data.

References

[1] Angel Aguiar, Maksym Chepeliev, Erwin Corong, Robert McDougall, and Dominique van der Mensbrugghe. The GTAP Data Base: Version 10. *Journal of Global Economic Analysis*, 4(1):1–27, Jun 2019.

[2] Maksym Chepeliev. GTAP-Power 10a Database: a Technical Note. Research Memorandum No. 31. Available at https://www.gtap.agecon.purdue.edu, 2020.

[3] Curt Covey, Krishna M. AchutaRao, Ulrich Cubasch, Phil Jones, Steven J. Lambert, Michael E. Mann, Thomas J. Phillips, , and Karl E. Taylor. An Overview of Results from the Coupled Model Intercomparison Project (CMIP). Program for Climate Model Diagnosis & Intercomparison. Available at https://pcmdi.llnl.gov/mips/cmip/, 2003.

[4] Esri-USGS. ArcGIS Data Store. ArcGIS Enterprise. Available at https://hub.arcgis.com/datasets/, 2022.

[5] FAO. Average daily dietary energy consumption per capita. Food and Agriculture Organisation. Available at http://www.fao.org/, 2020.

[6] FAO. Calculation of the Energy Contents of Foods- Energy Conversion Factors. Food and Agriculture Organisation. Available at http://www.fao.org/, 2020.

[7] FAO. FAO Stats. Food and Agriculture of the United States (FAO) Statistics. Available at http://www.fao.org/faostat/en/, 2020.

[8] FAO. FAO Statistics. Food and Agriculture Organisation of the United Nation. Statistics. Available at https://www.fao.org/faostat/en/data/QCL, 2022.

[9] Günther Fischer, Francesco N.Tubiello, Harrij van Velthuizen, and David A.Wiberg. Climate change impacts on irrigation water requirements: Effects of mitigation, 1990–2080. *Technological Forecasting and Social Change*, 74(7):1083–1107, 2007.

[10] F Gassert, M. Landis, M. Luck, P. Reig, and T. Shiao. Aqueduct Global Maps 2.1. Working Paper. Washington, DC: World Resources Institute. Available at http://www.wri.org/publication/aqueduct-metadata-global., 2014.

[11] F. Giunta, R. Motzo, and M. Deidda. Effect of drought on yield and yield components of durum wheat and triticale in a mediterranean environment. *Field Crops Research*, 33(4):399–409, 1993.

[12] GTAP. GTAP (Global Trade Analysis Project). Global Trade Analysis Project. Available at https://www.gtap.agecon.purdue.edu/., 2021.

[13] IIASA. SSP Database (Shared Socioeconomic Pathways) - Version 2.0. International Institute for Applied Systems Analysis, Online, Available at https://tntcat.iiasa.ac.at/SspDb, Accessed date: 12 March 2019, 2019.

[14] Haqiqi Iman, Farzad Taheripour, Jing Liu, and Dominique van der Mensbrugghe. Introducing Irrigation Water into GTAP 9 Data Base. GTAP Resources 5168. Available at https://www.gtap.agecon.purdue.edu, 2016.

[15] IPCC. Climate change 2021: The physical science basis. Contribution of Working Group I to the Sixth Assessment Report of the Intergovernmental Panel on Climate Change [Masson-Delmotte, V., P. Zhai, A. Pirani, S. L. Connors, C. Péan, S. Berger, N. Caud, Y. Chen, L. Goldfarb, M. I. Gomis, M. Huang, K. Leitzell, E. Lonnoy, J. B.R. Matthews, T. K. Maycock, T. Waterfield, O. Yelekçi, R. Yu and B. Zhou (eds.)]. Cambridge University Press., 2021.

[16] T. Kjellström, R.S Kovats, S.J Lloyd, T. Holt, and R.S.J Tol. The direct impact of climate change on regional labor productivity. *Archives of Environ. Occup. Health*, 64(4):217–27, 2009.

[17] T Kompas, V. H Pham, and T. N Che. The effects of climate change on GDP by country and the global economic gains from complying with the Paris Climate Accord. *Earth's Future*, 6(8):1153–73, 2018.

[18] Tom Kompas and Pham Van Ha. The 'curse of dimensionality' resolved: The effects of climate change and trade barriers in large dimensional modelling. *Economic Modelling*, 80:103–110, 2019.

[19] Huey-Lin Lee. Incorporating agro-ecologically zoned land use data and landbased greenhouse gases emissions into the GTAP framework. Research Publication. Available at https://www.researchgate.net/publication/, 2004.

[20] Matt Luck, Matt Landis, and Francis Gassert. Aqueduct Water Stress Projections: Decadal Projections of Water Supply and Demand Using CMIP5 GCMs. World Resource Institute. Available at https://resourcewatch.org/data/, 2022.

[21] Mirza Faisal Qaseem, Rahmatullah Qureshi, and Humaira Shaheen. Effects of Pre-Anthesis Drought, Heat and Their Combination on the Growth, Yield and Physiology of diverse Wheat *(Triticum aestivum L.)* Genotypes Varying in Sensitivity to Heat and drought stress. *Nature Briefing*, 9(1):1–12, 2019.

[22] Roberto Roson and Martina Sartori. Estimation of climate change damage functions for 140 regions in the gtap 9 database. *Journal of Global Economic Analysis*, 1(2):78–115, 2016.

[23] Victor O. Sadras, Francisco J. Villalobos, Francisco Orgaz, and Elias Fereres. Effects of Water Stress on Crop Production. GreenFacts. Available at https://www.greenfacts.org/, 2017.

[24] Karl E. Taylor, Ronald J. Stouffer, and Gerald a. Meehl. An Overview of CMIP5 and the Experiment Design. *Bulletin of the American Meteorological Society*, 93:485–98, 2012.

[25] WRI. Aqueduct Water Stress Projections. World Resource Institute. Available at https://resourcewatch.org/data/, 2022.

[26] WRI. Geospatial Datasets. World Resource Institute. Available at https://datasets.wri.org/dataset/, 2022.

[27] Wenhui Zhao, Leizhen Liu, Qiu Shen, Jianhua Yang, Xinyi Han, Feng Tian, and Jianjun Wu. Effects of Water Stress on Photosynthesis, Yield, and Water Use Efficiency in Winter Wheat. *Water*, 12(8):1–19, 2020.
